# Customer retention and churn prediction in the telecommunication industry: a case study on a Danish university

**DOI:** 10.1007/s42452-023-05389-6

**Published:** 2023-06-03

**Authors:** Sarkaft Saleh, Subrata Saha

**Affiliations:** Department of Materials and Production, Aalborg University, 9220 Aalborg East, Denmark

**Keywords:** Customer retention, Churn prediction, Classification algorithms, Telecommunication.

## Abstract

In this study, we explore the possible factors affecting churn in the Danish telecommunication industry and how those factors connect with retention strategies. The Danish telecommunication industry is experiencing a saturated market regarding the number of customers, but the number of service providers has increased significantly in recent years. Due to the high costs of acquiring new customers, the telecommunication industry put great emphasis on retaining customers in such an intensely competitive industry. We employ five machine learning algorithms: random forest, AdaBoost, logistic regression, extreme gradient boosting classifier, and decision tree classifier on four datasets from two geographical regions, Denmark and the USA. The first three datasets are from online repositories, and the last one contains responses from 311 students from Aalborg University collected through a survey. We identify key features extracted by the best-performing algorithms based on five performance measures. Based on that, we aggregate all the features that appear important for each dataset. The results demonstrate that customers’ preferences are not aligned. Among the prominent drivers, we find that service quality, customer satisfaction, offering subscription plan upgrades, and network coverage are unique to the Danish student population. Telecommunication companies need to integrate the sociohistoric milieu of the Nordic countries to tailor their retention policies to different consumer cultures.

**Supplementary Information:**

The online version contains supplementary material available at 10.1007/s42452-023-05389-6.

## Article highlights

(i) Five machine learning algorithms were used on four datasets to extract the key factors reflecting the preferences of customers in two regions.
(ii) Unique churn prediction and customer retention strategies are necessary for each region.
(iii) Network coverage, customer satisfaction, service quality and subscription upgrades affect Danish students’ churn.

**Supplementary Information:**

The online version contains supplementary material available at 10.1007/s42452-023-05389-6.

## Introduction

Customer churn is a common problem across businesses in numerous industries, including finance [1], news [2], insurance [3], online mobile gaming [4], telecommunication [5], and online gambling [6]. According to [7], *churn management is the concept of identifying those customers who intend to move their custom to a competing service provider*. Customers might stop using a product or service for different reasons—some that might be inevitable, and others might not. Therefore, predicting which customers are likely to churn and the corresponding factors associated with their preferences are crucial to protect recurring revenue, enhance customer retention, and ensure growth [8, 9].

In the telecommunication industry, research on customer churn analysis has become increasingly important for identifying key factors affecting consumers [9]. Getting a new customer can cost five to twenty-five times more than keeping an existing customer [10]. Therefore, the industry has paid much attention to attracting new customers and retaining existing customers [11]. Empirical research also supports this; for instance, Bain & Company reported that increasing customer retention rates by 5% can increase profits from 25 to 95% [12]. Globalization and access to communication technology have resulted in multiple alternatives, motivating customers to switch from one service provider to another [13]. Due to high competition, the effects of regulatory pricing intervention and consolidation [14], and the growing popularity of over-the-top (OTT) services, the telecommunication industry faces shrinking revenues and profitability. Accurate prediction assists managers in identifying necessary actions to be incorporated into their customer relationship management (CRM), such as whether to improve the service experience, design proactive campaigns to boost adoption, or re-engage at-risk customers. Over the years, the churn prediction problem has been studied from different perspectives, such as defining churn in different contexts [15], identifying key factors affecting customers [16], developing new algorithms to improve performance accuracy [17], and reducing expenses for win-back campaigns [18].

Predicting churn manually by considering many different aspects is not straightforward, and researchers use AI solutions to automate and scale churn prediction. Churn prediction models are developed to detect customers with a high propensity to attrite [19, 20]. Many supervised machine learning algorithms (MLAs), convolutional neural network (CNN) models, and text-mining algorithms are employed to analyse churn in the telecommunication sector [5, 17]. Depending on the features processed in the algorithms, researchers group the models into either static-based or dynamic-based [15]. By obtaining static features representative of the customer of a telecommunication service, we analyse the static behaviour of the customers.

With world-leading purchasing power, the Nordics are among the most attractive regions for telecommunication service providers. More than 26 service providers have a strong presence due to a higher penetration rate in Denmark. However, in recent years, the total revenue in this industry has remained stagnant. Evidence shows that some companies are either merged with others or acquired by others [21], (e.g., TDC acquired Plenti in 2017 and eesy A/S in 2020 [22]). Therefore, we aim to explore the following research questions: *What are the key factors affecting customer churn in the Danish student community? Which aspect(s) of the Danish telecommunication industry need more prioritization to prevent customer churn?* We used four datasets for the evaluation: three are from online repositories, namely IBM Telco, Maven Telco, and Cell2Cell; and a new dataset was collected through questionnaires from a Danish university. Five MLAs are used, and the best-performing MLAs are identified based on five performance measures for each dataset. The key features are aggregated to find similarities and distinctions between the two geographical regions. The key contributions of the present study are as follows: first, consumer preferences in the Danish telecommunication indutry (DTI) have not yet been explored [9]. Therefore, this study provides insights into how customer retention policy might differ in the Nordic region. The feature of discriminating preferences can help companies to build strategic planning. Moreover, the dataset containing opinions from the Danish student population could help as a reference for further enhancement in designing churn prediction theory. Second, we identify some of the retention strategies implemented by Danish telecommunication companies. We supplement with the factors such as subscription plan offers, service quality, customer satisfaction, and network coverage to retain consumers’ needs to be emphasized more in the context of Denmark.

The remainder of the study is organized as follows: In the following subsection, we present literature review and overview of the DTI. Section 2 describes four datasets and the methodologies used in this study. Section 3 presents all the results regarding MLA performance and the key features affecting churn. In Sect. 4, managerial insights are presented regarding the key difference between the DTI and other regions and how those relate to CRM. In Sect. 5, final remarks and future extension areas of the proposed study are presented.

### Customer retention strategy and churn analysis

According to [23], *CRM is the strategic process of selecting customers that a firm can most profitably serve and shaping interactions between a company and these customers. The ultimate goal is to optimize the current and future value of customers for the company*. The key idea is that strategic decisions ensure improved customer satisfaction. In return, such measures result in an increase in customer retention and maintain stable growth. The literature on CRM emphasizes the use of relationship marketing strategies to identify and understand customer needs [10, 24]. Therefore, it is crucial to understand the existing customers and their needs and expectations through their purchasing patterns and sensitivity to price and loyalty programs. Studies report that a list of different activities affects and improves customer retention in the telecommunication industry, such as (i) loyalty activities [11, 25], (ii) customer satisfaction activities [26, 27], and (iii) service quality [28, 29].

Churn analysis and CRM are two sides of the same coin: the former determines the factors that cause people to leave, and the latter focuses on increasing the overall value of its customer base [30, 31]. In regard to churn analysis, Verbeke et al., [19] analyses the churn problem by applying data mining techniques (ALBA & AntMiner+) to generate accurate and comprehensible rule sets instead of a particular threshold. Note that the key performance indicator (KPI), such as profit, is also an important issue in evaluating strategies. In this context, [32] presents a profit measure for churn models, which selects a fraction of customers to include, leading to a significant increase in profits compared to traditional statistical measures. Moreover, the findings show that oversampling does not significantly improve performance. Later, the authors introduce a new expected maximum profit (EMP) criterion, which supports businesses in selecting a model that maximizes the profits and the fraction of the customer base to include in retention campaigns [33]. The implication of social network analysis models is also tested to predict customer churn [34]. Moreover, researchers also evaluated the EMP measure to compare algorithms for addressing class imbalance in churn prediction [35]. An overview of some recent studies conducted in the telecommunications industry is listed in Table 1.

**Table 1:** Literature review of churn prediction in telecommunication industry

Study	Dataset(s)	Method(s)	Evaluation metric(s)	Key remark(s)
[36]	IBM telco & Cell2Cell	Deep-BP-ANN, LR,	Acc., AUC, Recall	Deep learning method outperform existing
		NB, KNN & XGBC	Precision & F1-Score	ML methods in performance measures.
[5]	IBM telco & Cell2Cell	KNN, RF, LR, NB, GBC,	Acc., Precision	Cell2Cell: RF, NB, XGBC & AdaBoost.
		XGBC, AdaBoost & ETC	Recall & F1-Score	IBM: LR, NB & XGBC
				A proposed combination of classifiers out-
				perform all in terms of accuracy.
[13]	Cell2cell & Orange	DL, NB, DT, GBC, RF	Acc., Precision,	Ensemble classifiers out-
			Recall & F-score	performs the single classification techniques.
[17]	IBM telco	CNN	Accuracy	Proposed CNN model support businesses
				to predict CC by TPR of 95%.
[37]	Larose Telco, Telecom1,	LDA, SVM, RF,	Acc., RMSE, KS,	Projection pursuit RF perform better pre-
	Cell2cell, IBM telco,	Project-pursuit RF	AUC, Precision, Sens.,	dictive than utilised methods.
	Telcom2, Asian Telco		Spec., Gini, OOB	
			& F-score	
[38]	Dataset-1, IBM telco,	Bayesian binomial test	Precision, Recall,	Non-churn customers and churn have resem-
	PAKKD & Dataset-4	Distance factor	F-measure & Acc.	bling features which negatively effects
				classification failure rate, therefore, focus
				on level of certainty is crucial to consider.
[16]	Syria-Telco	XGBC, GBM-Tree, DT,	AUC	XGBC outperform all algorithms
		RF		presenting 93.301% AUC.
[39]	Cell2cell	DT, ANN, SVM, kNN	AUC, Acc., Sens.,	Combining SOM with classifier ensembles by
			Spec. & F-measure,	PCA provide best prediction results.
				Filtering out irrelevant features is im-
				portant for data pre-processing.
[40]	Dataset-1	4 FUZZY classifiers &	TP & FP rate,	VQNN (FP rate) and OWANN (TP rate &
		9 NON-FUZZY classifiers	AUC	AUC) FUZZY classifier outperform all.
[41]	UCI repository	SVM (Kernel = RBF, LIN,	Acc. & Gain	SVM(POL) perform best with 88.56%
		POL & SIG)		accuracy.
[42]	Taiwan-Telco	DT & BP-ANN	Lift & Hit-Ratio	BP-ANN outperform DT

*ACC* accuracy, *ANN* artificial neural network, *AUC* area under the curve, *BP-ANN* back propagation artificial neural network, *CC* customer churn, *CNN* convolutional neural network, *DL* deep learning, *ETC* extra tree classifier, *GBC* gradient boosting classifier, *GBM* gradient boosting machine, *KNN* k nearest neighbor, *KS* kolmogorov-smirnov test, *LDA* linear discriminant analysis, *LIN* linear,*LR* logistic regression, *NB* naive bayes, *OOB* out-of-bag error, *OWANN* ordered weighted average nearest neighbour, *PCA* principal component analysis, *POL* polynomial, *RBF* radial basis function, *RF* random forest, *RMSE* root mean squared error, *SENS* sensitivity, *SIG* sigmoid, *SOM* self-organizing map, *SPEC* specificity, *SVM* support vector machine, *TPR* true positive rate, *VQNN* vaguely quantified nearest neighbors, *XGBC* extreme gradient boosting classifier

Table 1 demonstrates the selection of algorithms and that their efficiency in identifying key features is not unique [8, 43, 44]. Researchers used freely available repository datasets: IBM Telco and Cell2Cell, to evaluate the performance of MLAs (e.g., XGBC, DT) by benchmarking different performance measures such as accuracy, F1-score, precision, and others. However, the datasets used in Table 1 are mostly from a particular geographical region. It is certain that acquiring data requires both time and expense. However, based on the data of a specific area, we cannot obtain a thorough overview of preferences for customers from other regions. Due to lifestyle, social structures, regulations, and other factors, customers’ preferences might differ considerably. To our knowledge, the preferences of Danish student population are missing.

### Overview of the DTI

**Fig. 1:**
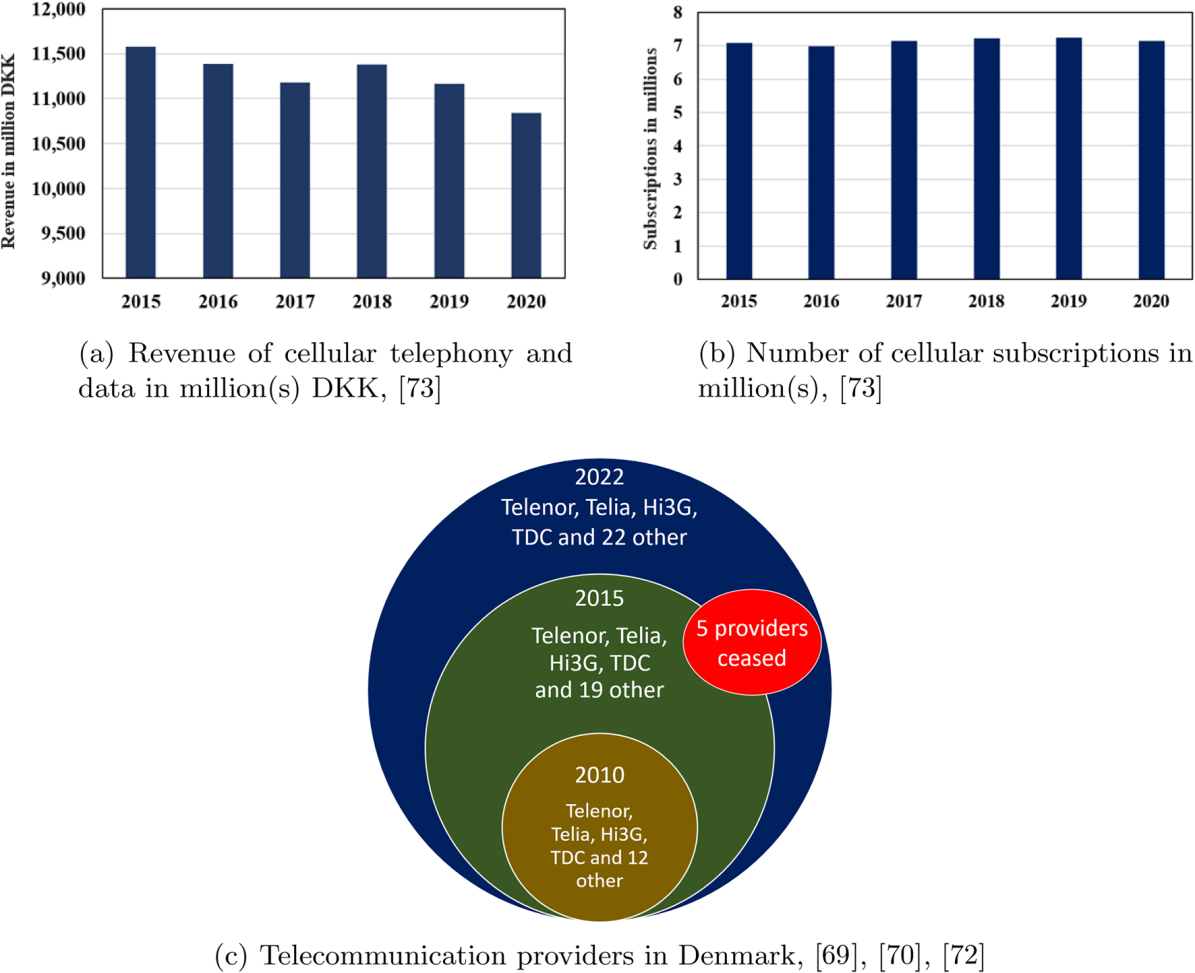
Characteristics of the DTI

In a recent survey, Bhattacharyya et al. [9] provide a global overview of the studies in the field of churn prediction. As reported by the authors, churn prediction problems from the perspective of the DTI  is sparse. The industry in Denmark has experienced a static market regarding the number of mobile cellular subscriptions from 2015 to 2020 (Fig. 1b). However, during that period, the number of registered telecommunication companies increased, and currently, there are 26 service providers. Telenor, Telia, Hi3G, and TDC remain market leaders in terms of revenue or number of subscribers. Some small-scale, low-cost service providers also exist (e.g., GreenTel, dukaTALE). Their pricing and promotion strategies might impact the gross revenue of the whole industry. The following Fig. 1 present an overview of total revenue in the telecom industry, subscriptions, and the number of service providers in Denmark.

Therefore, it is highly challenging for Danish companies to maintain stable financial growth due to increasing competition. In this regard, predicting the key factors influencing churn is crucial for them. As shown in Fig. 1c, the DTI has experienced increasing growth in the number of service providers. After the COVID-19 pandemic, people are more familiar with OTT media services, and their subscriptions affect gross revenue. Moreover, telecommunication service providers utilise substantial fixed and shareable infrastructure that must be offset by revenue. The population growth in Denmark over the past ten years has remained stagnant [45] and acquiring new customers is a challenging issue. Identifying factors affecting churn can benefit telecommunication service providers to retain customers by minimizing customer churn. Therefore, our study can provide insights into the key factors to be emphasized for future business.

## Methodology

### Data used for the evaluation

The data used in this study consist of three online available repository datasets: IBM Telco, Cell2Cell and Maven Telco [5, 36]. A survey was conducted to collect responses of students from Aalborg University, because no online repository data are available in reflecting Danish customer preference in the telecommunication industry. Therefore, the survey data can reflect student preferences in this country. We designed our questionnaire to collect data on four categories: (i) customer care details, (ii) customer demography, (iii) payment methods, and (iv) value-added services. The survey was a mixed-method survey; some questions required quantitative answers and qualitative responses. Each dataset is described below:

**IBM Telco data** are provided by International Business Machines (IBM) Corporation and extracted from the Kaggle Data Platform [46]. The dataset consists of 7043 samples with 21 attributes. In addition, it includes information about customer churn and three numeric features, such as tenure period, the monthly and total charges from each customer. The categorical features includes for each customer information about service attributes (internet service, security, device protection, streaming TV, etc.), information about the customer account (type of contract, payment method, etc.), and demographic information about customers (gender, age, etc.). The IBM Telco dataset is imbalanced, consisting of 1869 churned customers and 5174 nonchurned customers registered in California, USA.

**Maven Analytics Telco churn data** are sourced from IBM and extracted from the Maven Analytics Data Ground [47]. The dataset is constructed with attributes similar to the IBM telco dataset but supplemented with additional features such as average monthly GB download, unlimited data option, age, streaming music, long distance charges, etc. The full list of features are listed in 38 columns. The dataset has been cleaned for missing values or empty features, and customer status is restricted to either *churned* or *stayed*. Similarly, with the IBM Telco dataset, samples are from customers in California, USA. Maven Telco dataset consist of 1586 churned customers and 3015 nonchurned customers.

**Cell2Cell data** are from the Teradata Center for CRM of Duke University USA and extracted from the Kaggle Data Platform [48]. The dataset consist of 71,047 samples and 58 attributes. The dataset contains information about customers including (i) value-added services, (ii) demographic information, (iii) payment and bill method, (iv) usage patterns. The samples are from one of the largest wireless companies in the USA. Cell2Cell data is imbalanced, which consists of 14,257 churned customers and 35,519 nonchurned customers registered in USA.

**AAU data** are collected through a survey carried out at Aalborg University (AAU), Denmark. A total of 311 students participated in the survey, and 288 provided complete feedback based on 21 questions (the number of questions for each respondent varied according to the answers). The authors designed the survey questionnaire based on sixteen quantitative and five qualitative questions. For instance, we acquire responses against the question: *Which of the following criteria do you find the most important while choosing a service provider?*, where the respondent could choose among one or several options: price, streaming services, data GB for roaming, online services (protection, back-up), high data GB, professional brand, call hours for roaming, promotional offers, or other. The respective office assistants sent the survey questionnaire to undergraduate/postgraduate students in Materials and Production, Energy, Architecture, Design, & Media Technology, and Politics & Society departments. The aim of involving the office assistants was to ensure anonymity and allow respondents to share their thoughts truthfully. A five-point Likert scale was used so students could choose from two extremes, two intermediates, and one neutral option. Excluding incomplete responses, the dataset consisted of 109 churned samples and 179 nonchurned samples. We refer to Table 2 for an overview of the four datasets. Additionally, we refer to Tables S2.1 and S2.2 in the Supplementary file for the description of features for IBM Telco data, Maven Telco data, and Cell2Cell data.

**Table 2:** Datasets after exclusion of irrelevant features and missing values

Dataset (Version)	Region	No. of features	Sample size	No. of churned customers (%)
IBM Telco	USA	19	7043	1869 (26.5)
Maven Telco	USA	27	4601	1586 (34.5)
Cell2Cell	USA	39	49,776	14,257 (28.6)
AAU	Denmark	18	288	109 (37.8)

### Machine learning algorithms

It is extremely challenging to prepare a proactive retention strategy if the methodology inaccurately identifies the key factors affecting customer churn. Otherwise, it can mislead managers and require them to change their action plan. The performance measures of each MLA can be very different when implemented in different datasets for many reasons, such as hyperparameter settings, dataset segmentation, and computation power. Therefore, their feature importance might also not be aligned. The permutation feature importance technique is utilised in this study using Python by importing the Scikit-learn module; *permutation feature importance*. The technique is defined as the relationship between the feature and the target. A drop in the model accuracy indicates how much the developed model depends on the specific feature. Therefore, we rely on the outcomes of five algorithms to aggregate important features using the Python module Scikit-learn. We applied synthetic minority oversampling technique (SMOTE) to the AAU data to balance and analyse the difference [49, 50]. In real-world data, the majority of samples belong to one class (*nonchurners*), while the more important class is typically the minority group (*churners*). Previous studies have shown that applying MLAs to large imbalanced dataset tend to yield poor performance [49, 50]. The SMOTE is widely used in the field of churn prediction to overcome this challenge. It generates artificial data for the minority class for each original minority sample while not taking into account the instances from the majority class [35]. This can result in not representing the real-world characteristics of a dataset, hence, the outcome may not reflect the true nature of the minority class. We refer to Section S5 in the Supplementary file for details.

**Random forest (RF)** is an effective classification method with nonlinear data. A number of decision trees are created by choosing any random sample of attributes from the predictor attribute set. The final decision tree mainly uses weighted averages for the predictions. Unlike other algorithms, it performs better if correlated features exist in the data [51]. RF is utilised for churn prediction by [5, 18].

**Adaboost Classifier (ADA)** is an ensemble classifier that iteratively retrains the algorithm by selecting the training set based on the effectiveness of prior training. While a single algorithm might not be very effective at classifying objects, the overall classifier can achieve a high accuracy score when paired with a boosting ensemble algorithm. It enhances the algorithm’s prediction performance by turning a group of weak learners into strong learners [51]. ADA is used for churn prediction by [5, 52].

**Logistic regression (LR)** uses the logistic function to squeeze the output rather than fitting it to a straight line or hyperplane. The function does not have a linear relationship with weights. A probability is created from the weighted sum using the logistic function. It uses a maximum-likelihood estimator to maximize the probability of the observed outcome. It computes the error for each prediction and the prediction value for each instance in the training set. This procedure repeats until the model is sufficiently accurate [51]. LR algorithm is used for churn prediction by [5, 36].

**Extreme gradient boosting classifier (XGBC**) is a decision tree-based ensemble technique created using gradient boosting. The objective function minimized by XGBC combines a penalty function for model complexity with a loss function. The gradient boosting method first computes the residuals of the previously applied model using new models, and then it combines both of these results. It is known as “gradient boosting” because it minimizes loss when introducing new models by employing a gradient algorithm [53]. XGBC algorithm is used by [5, 36] for churn prediction.

**Decision tree (DT)** is constructed as a tree by adding nodes along each branch until it reaches a terminal node. Each node in a decision tree is given a class label and represents a test on its attributes. The branches descending from each node in the DT show all potential values that an attribute may take. The advantages of decision tree algorithms are that they are quite resistant to noise and are relatively simple to interpret [51]. DT is used in recent studies for churn prediction [13, 39].

### Performance measure

Five measures are evaluated for each algorithm to find the best-performing. The accuracy metric ($Accuracy = \frac{TP + TN}{TP + TN + FN + FP}$) is used to measure the total number of correctly identified instances. The precision metric ($Precision = \frac{TP}{TP + FP}$) is used to measure how the model is observing the actual number of positives against the predicted positive. The negative predictive value ($NPV = \frac{TN}{TN+FN}$) is used to measure how the model identifies the non-churners. F1-score ($F1-score = \frac{2TP}{2TP + FP + FN}$) is used to measure how accurate the algorithm’s performance is. Finally, area under the curve (AUC) is calculated by ($AUC = \frac{1}{2}- \frac{FP}{2(FP+TN)}+\frac{TP}{2(TP+FN)}$), where *TP* true Positive; *TN* true Negative; *FP* false Positive; and *FN* false Negative. A higher result indicates a more accurate performance. In addition, top-decile lift is measured for best performing MLAs for each dataset.

## Results

**Fig. 2:**
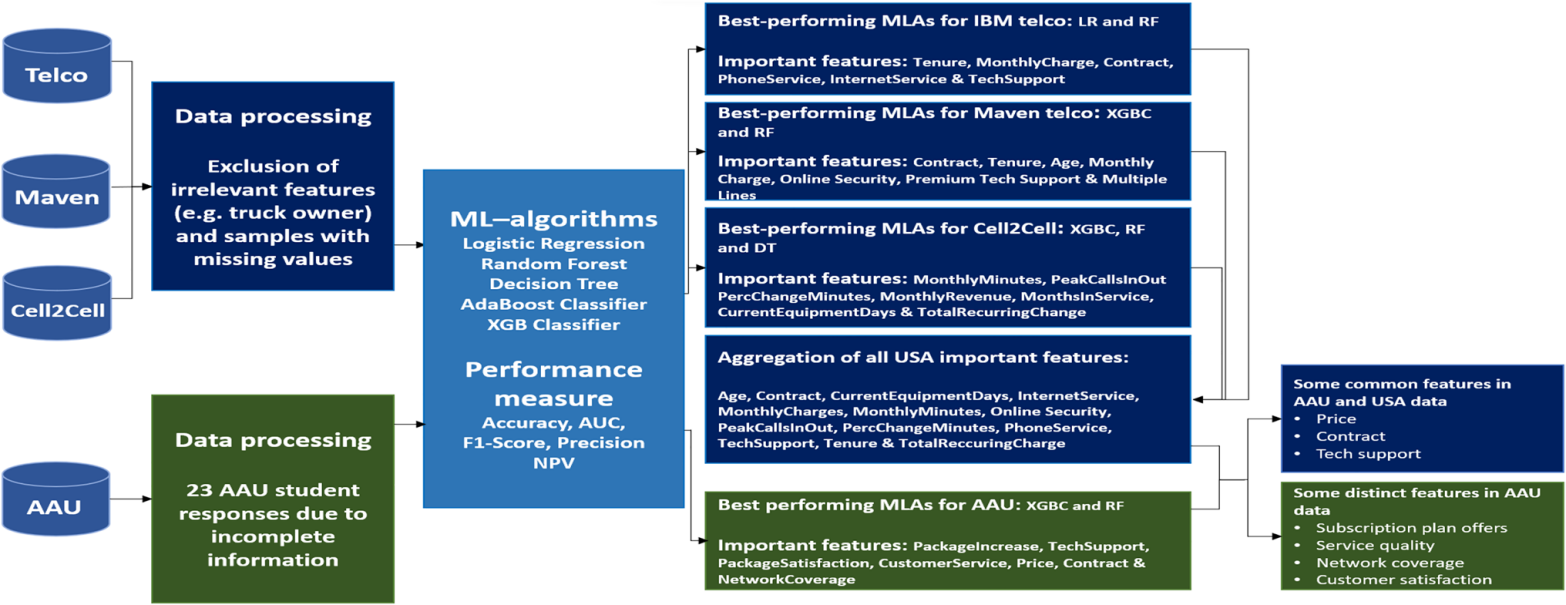
Computational scheme for study and key features

Before implementing MLAs, we conduct preanalysis to exclude samples with missing values and features that are not relevant (e.g., we exclude features such as *marital status* for the Cell2Cell dataset). We refer to Section S2 in the Supplementary file for the detailed features we considered for the analysis. Each dataset is divided into two subsets: a training set and test set with a 70/30 ratio. The first subset is used to fit the algorithms, and the test set is used to predict unseen data. We refer to Table S4.1 in the Supplementary file for the details of the parameter settings used to implement each algorithm. Furthermore, the best-performing MLA(s) based on each performance measure was determined for each dataset. Figure 2 presents the computational scheme used and the results obtained in this study.

**Fig. 3:**
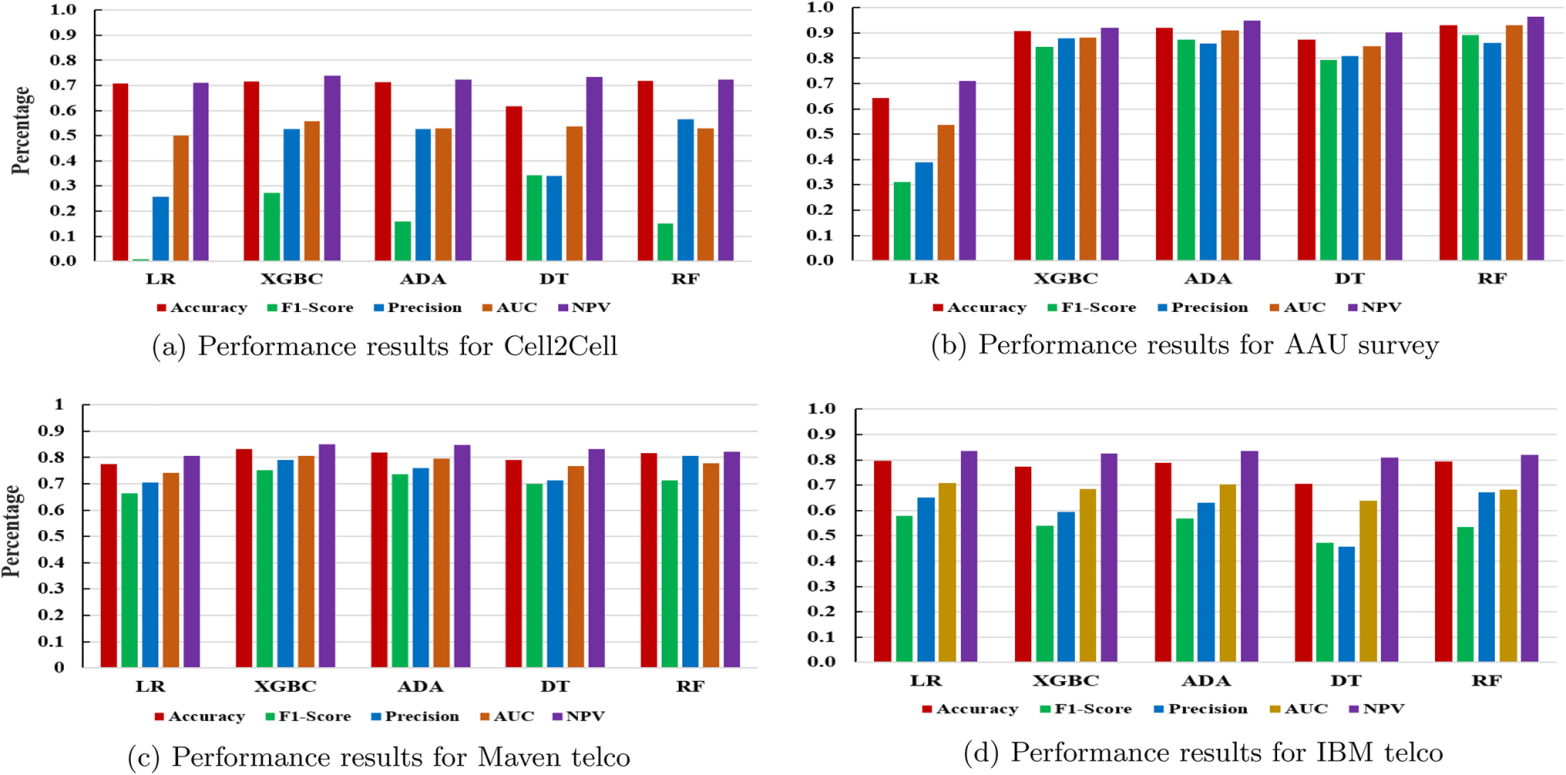
Performance results of the test set based on five different measures

From Fig. 2, we find that the key features for each classification algorithm are not aligned but not wholly different. For clarity, we present the performance measure for each MLA in four datasets in Fig. 3.

We refer to Fig. 3 demonstrate that in terms of each performance measurement, LR (Acc., F1-score, AUC & NPV) and RF (Precision) outperformed other MLAs and are the best-performing algorithms for IBM Telco dataset. Similarly, for the Maven dataset, XGBC (Acc., F1-score, AUC & NPV) and RF (Precision) outperformed other utilised MLAs. For the Cell2Cell dataset, XGBC (Acc., AUC & NPV), RF (Acc. & precision) and DT (F1-score) outperformed other MLAs. Finally, for the AAU dataset, RF (Acc., F1-score, AUC & NPV) and XGBC (Precision) outperformed the other implemented MLAs. Furthermore, Tables S4.1 to S4.4 in the Supplementary file present the top-decile lift for the best-performing algorithms for each dataset. The top-decile lift focuses exclusively on the most critical group of customers and their churn risk. Gain shows the percentage of actual churners covered at a given decile level, whereas lift indicates the ratio percentage to the random rate at a given decile level. As shown in Table S4.3 in the Supplementary file, in decile level 4, XGBC obtains 43.8% of churners covered in the top 40% of the data. A lift of 1.1 indicates that in 40% of the data based on the model, we could most likely find the churners 1.1 times more than randomly selected 40% of the data without a model. We apply the SMOTE technique to balance the two classes of the AAU dataset. Although we observe an increase in performance in some algorithms, we obtain no changes in the best-performing MLAs based on five different performance measures (Table S5.1 in Supplementary file). Nevertheless, XGBC and RF outperformed the other MLAs, and their feature importance is presented in Table S5.2 in the Supplementary file. We observe mostly similar important features for the balanced/imbalanced dataset with *PaymentMethod* included and *PackageSatisfaction* and *CustomerService* excluded for the latter. As we obtain significantly high performance without data manipulation by applying data balancing techniques (see Table S3.1 in Supplementary file), we present our conclusion based on those unbalanced datasets to avoid any recommendation with synthetic data samples included.

The result supports that the key features identified by the MLAs are not aligned. For example, if we look at the important features obtained through the AdaBoost algorithm, which is also popular in churn analysis for telecommunications data [52], the key features are significantly different compared to those identified by other algorithms. Noticeably, the decision tree algorithm performs poorly compared to all four other algorithms in our study (see Table S3.1). Similarly, the LR algorithm is outperformed by the other four algorithms in the Maven, Cell2Cell, and AAU datasets. Therefore, the five algorithms’ implementations help us aggregate possible features in two geographical areas. Note that we can exclude the LR algorithm if we only perform churn analysis based on three datasets. We also found that LR identified *brand* as a key feature, which was not recognized by the other algorithms. Note that, in the telecommunication industry, *brand* is also a key [54].

**Fig. 4:**
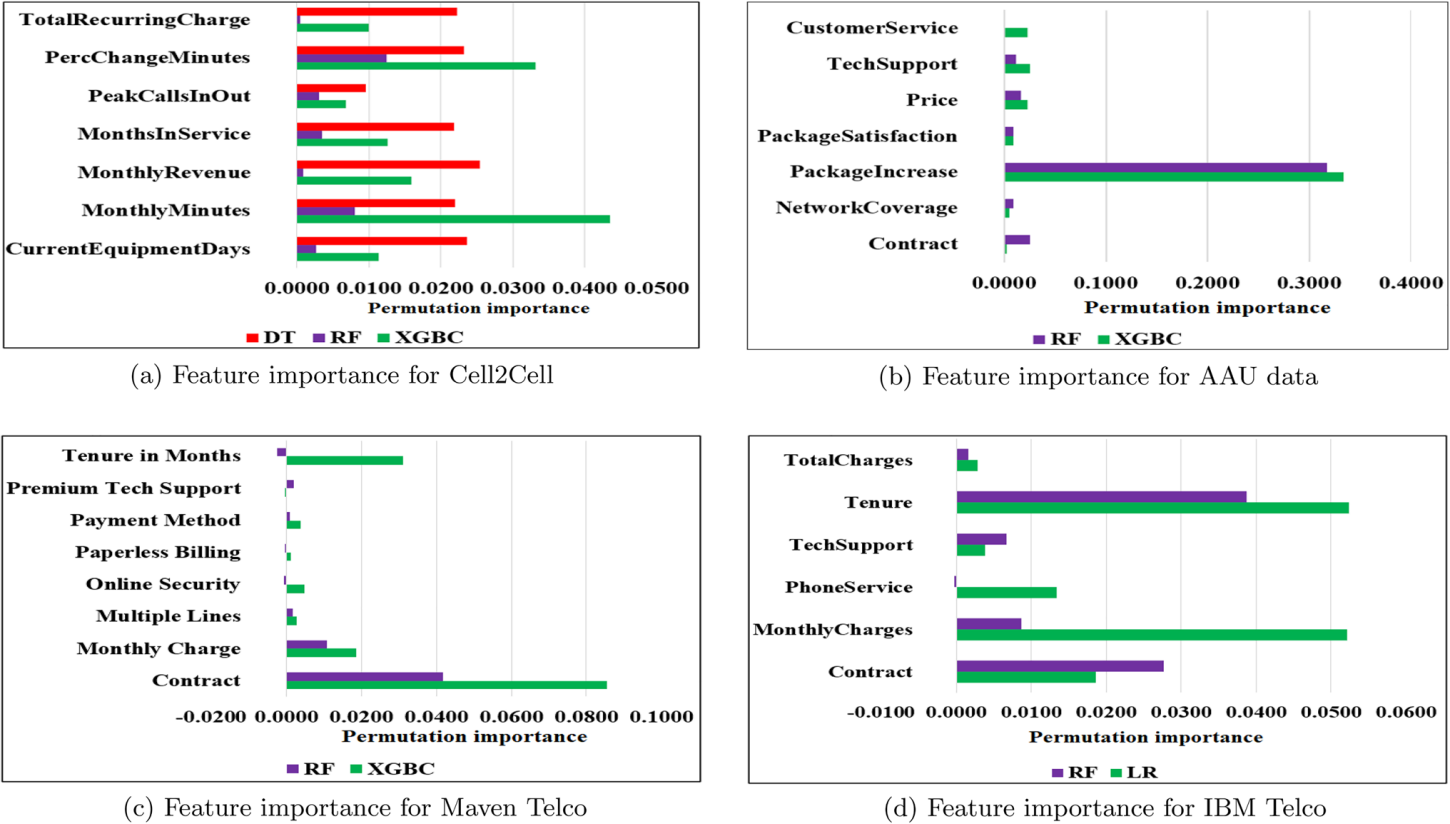
Feature importance from best-performing algorithms for each dataset

Figure 4 presents the top features affecting customers to churn for each dataset from the best-performing algorithms and their importance. In Tables S3.4–S3.7, the numerical values present all features used to predict churners. The higher the value, the more important that specific feature is to the churn risk. The values indicate how much each algorithm depends on that particular feature to identify the churners from each dataset. For instance, Fig. 4c demonstrates that contract type and the monthly charge impact customer churn most. Table S3.3 in the Supplementary file presents each algorithm’s top five features affecting customer churn.

Recall that Fig. 2 presents six unique features from the IBM Telco dataset, seven from the Maven Telco dataset, seven from the Cell2Cell dataset, and seven for the AAU dataset when combining features from the best-performing MLAs. Aggregating features that appear significant in datasets from the USA, we obtain a list of 13 unique features. Some of these features appear as key in the context of USA customers but not for AAU respondents. Some features such as *Age, CurrentEquipmentDays, InternetService, Online Security, PhoneService* and *PeakCallsInOut*, are not directly related to the AAU dataset because they have not been found to be relevant or due to the lack of data availability. For instance, we did not consider age as a feature in the AAU dataset due to a focus only on undergraduates/postgraduates in the survey. Since most Danish companies provide additional data, the *InternetService* feature is not applicable to the DTI. However, in the USA, approximately 10% of mobile phone subscribers do not subscribe to wireless internet access [55]. The AAU survey includes other features such as PhoneService and OnlineSecurity (Fig. S1.2b), which were prioritized by most of the respondents. However, compared to price, data usage, and other factors, these features were given lower priority. A list of features appears important for the DTI but not for the USA’s telecommunication industry. Due to the easy access of other EU nations, network coverage, that is, accessibility of the internet and calling in other countries, becomes key as the participants are students enrolled from different countries (refer to the question in Fig. S1.3 in Section S1 in Supplementary file). For instance, network coverage is identified as *RoamingCalls* and *non-US travel* in Cell2Cell data but does not appear as important when we see the best-performing algorithms. *PackageIncrease* feature is included in questions (refer to Fig. S1.1j), and the motivation was to verify whether the respondent had been offered any promotion by their existing provider, such as an increase in subscription plan offers or other value-offering (e.g., gift certificate, online services included). This is identified in Cell2Cell data as *CustomerCareCalls* and *RetentionOffersAccepted*, but these features do not appear as key for the USA telecommunication industry. Moreover, questions in Fig. S1.3 present customer service and package satisfaction as features for AAU data. From USA data, these features are identified as *CustomerCareCalls*, *RetentionCalls* and *RetentionOffersAccepted*, which appears important only for the DTI.

We also identified key features that appear important in the AAU data as well as aggregated features in the other three datasets. For instance, the *Contract* feature appears important for IBM Telco and Maven Telco data, and this finding also emerges from AAU data. Similarly, we found that price features affect customer churn for Danish telecommunication, which also appears crucial for the USA industry for all datasets in terms of *MonthlyCharge*, *MonthlyMinutes* and *PercChangeMinutes*. We also found *TechSupport* to be essential in both the Danish and USA telecommunication industries.

## Discussion

Classification results demonstrate that a single MLA fails to ensure the best performance in all five performance measures. We found that two MLAs outperform other algorithms based on one or several performance measures for a dataset. These findings are well-suited to recent studies. For instance, [5] showed that LR ensured the highest F1-score (we also found LR outperformed based on accuracy and F1-score) for the IBM Telco dataset. Similarly, for the Cell2Cell dataset, [5] found XGBC to perform best in terms of accuracy and precision metrics (for accuracy metrics, we found XGBC and RF, and RF also ensured the highest in terms of precision). Therefore, we suggest that instead of relying on a single MLA, one should apply multiple MLAs to develop a set of important features.

We found some interesting areas: First, international network coverage appears important for students, but the number of respondents was too low. Since students are from a different region in the EU, outside calling facilities and internet usage are also key. This particular feature appears important, as shown in Fig. S1.2b and S1.3. Similarly, we found that five respondents switched as their parents switched. For them, parents were responsible for their subscriptions. In Europe, a parent sometimes pays the student’s subscription [56].

Analysing churn and initiating customer retention strategies has been a research priority in recent years [18]. In some studies, researchers only focus on either churn analysis [13, 36] or forming customer retention strategies without detailed background [57]. However, those are not separate. Therefore, we focus on the interlink between churn prediction and customer retention. As Jeff Bezos once quoted, *“We see our customers as guests to a party, and we are the hosts. It’s our job every day to make every important aspect of the customer experience a little bit better”*^1^. In this study, the findings of feature importance for the telecommunication industry on two geographically different datasets show both similarities and differences. However, information from the extracted datasets contains common features, e.g., Cell2Cell data are sourced from a telecommunication provider in the USA containing data about service usage such as call details and call duration, which we did not consider in the survey. For instance, we identified some important features for the USA regarding customer service usage, such as *PeakCallsInOut* and *PercChangeMinutes*. In the telecommunication sector, customers sometimes select the service provider based on their history and reputation. It might be a possible way to analyse key features based on each service provider. However, due to the sample size, it does not appear a feasible way to analyse AAU data. Similarly, with [58], we found that customer service (*customer care calls*) is not a key factor for the Cell2Cell dataset affecting customer churn. Nevertheless, we found it important in the context of the DTI. Similar to our findings, studies by [28] and [29] show that service quality greatly influences retention in the telecommunication industry. Additionally, we found that package satisfaction is an important feature in the Danish industry; similarly, with [56], the study presents that customer satisfaction affects the intent to continue service and is also observed in the Nordic region [54]. According to recent evidence, features such as price and satisfaction affect churn and are considered by business managers in the DTI. However, we also found that telecommunication companies operating in Denmark should consider implications such as upgrades in subscription plans and network coverage, as they affect customer churn. According to [59], existing customers in the Nordic region receiving discounts can be retained and are stimulated to purchase additional services from their existing provider more than customers without any discount offers.

In contrast to global telecommunication markets, e.g., the USA, China, or India, initiating customer retention strategies is especially crucial in the DTI. Customer retention is important for businesses experiencing saturated markets or lower growth of new customers as experienced in the DTI [60, 61]. The laws and regulations of the European Union have impacted the telecommunications industry in all markets of the European nations, as stated by the CEO of Vodafone Group [62]. Only three or four major mobile network operators (MNOs) serve a larger population on other markets in comparison to over 100 MNOs competing in the European market. In addition, the laws and regulations imposed on MNOs make it possible for mobile virtual network operators (MVNOs) to operate on their cellular networks. This has enabled telecommunications companies to exist with low costs and disrupt the telecommunication industry by solely competing on price, as observed in the DTI. Moreover, the laws and regulations of the EU prohibit major companies from merging, e.g., the merger of Telenor and Telia in 2015 was rejected to prevent them from forming a dominant company in the DTI [63].

In some recent initiatives, the major telecommunication companies in Denmark have shifted away from price-competing towards promoting campaigns with service attributes included in the subscriptions. For instance, Telia recently promoted a new brand strategy as one of the most expensive campaigns to offer cellular services, TV streaming, and internet as package deals targeted at families [64]. Telenor has recently shifted its focus away from highly competitive price offers to differentiate itself from the market by providing reassuring services such as safe internet browsing and screen switching. The result has been a success, presenting the best annual results in ten years and the highest quarterly satisfaction in the company’s history of operating in Denmark [64–67]. Tenure appears to be an important feature for US telecommunication in all three datasets in terms of *Tenure* or *MonthsinService* but does not appear to be important for Danish telecommunication. However, the question in Fig. S1.1f shows that we grouped the tenure period in monthly intervals, which are listed as units in months for USA data.

## Conclusion and future research

The DTI is experiencing saturation, and new strategies in addition to competing on price are necessary. Churn analysis and customer retention strategies are key in the highly competitive industry to predict churners and initiate proactive activities to retain existing customers. Five MLAs were implemented, and the performance was evaluated based on five different measures. The best-performing algorithms identified important features affecting customer churn in the telecommunication industry in the USA and Denmark. The results suggest that the DTI should upgrade subscription plan offers to retain existing customers and focus on service quality, customer satisfaction, and network coverage. We found that age is an important factor influencing churn [68], although we restricted our focus to undergraduate/postgraduate students. In the future, heterogeneity needs to be considered.

Theories of customer churn in the telecommunication industry are most often ingrained in the USA. However, the key factors affecting consumers might differ if investigated in a wider range of cultural and socioeconomic contexts. Therefore, to supplement recent efforts to develop more localized consumer culture and retention theory [69], we conduct churn analysis between two regions to explore more macro perspectives. The contribution has the potential to formulate conceptual dialogues for the telecommunication industry in the Nordic region, which is somehow aligned with the USA’s format but not utterly. We demonstrate new features that can be put on the springboard for strategy refinement. As we projected, a limitation of our survey is that we do not have evidence about the characteristics of the retention offers to Danish consumers and do not know whether targeted consumers accept them. The number of respondents for the AAU dataset represents the opinions of a particular age group, which might fail to provide an overview of the whole population. The number of respondents is also limited. There might be additional features, such as the effect of social networking and profitability, which can be included. The dataset also lacks call-detail records (CDRs) (such as the duration of calls or the frequency of communication between two customers). In future research, social network analytics may provide the opportunity to understand customer behaviour and further enhance churn prediction accuracy. One of the central challenges is accurately defining the social network based on available relational data, e.g., CDRs. Additionally, processing such large amounts of data from CDRs can be a difficult and time-consuming task as it often contains millions of records and requires considerable preprocessing before being able to utilise for analysis [34]. From the theoretical point of view, the static measure we used could be complemented by dynamic analysis, anticipating the possibility of proposing retention offers afterwards. However, the key features that discriminate telecommunication customers in two geographical regions can be considered a starting point for future evaluation and supplement churn prediction theory from a global perspective.

## Supplementary Information

Below is the link to the electronic supplementary material.

Supplementary file 1 (pdf 508 KB)

## Data Availability

All relevant data collected from Aalborg University are available on request from storage platform Data deposit at Aalborg University in the website: 10.57957/datadeposit.1fad02be-0b86-4281-bc5a-c92527172105 and S. Saleh.

## Footnotes

1 https://www.goodreads.com/quotes/794527-we-see-our-customers-as-invited-guests-to-a-party.
