# Supplementary material for: Customer retention and churn prediction in the telecommunication industry: a case study on a Danish university
Source: SN Appl Sci. 2023 Jun 3;5(7):173. doi: 10.1007/s42452-023-05389-6 (PMC10239051; doi:10.1007/s42452-023-05389-6)
Supplement: Supplementary file 1 — (pdf 508 KB) [file 42452_2023_5389_MOESM1_ESM.pdf]

Online Supplementary file for "Customer retention and churn prediction in the telecommunication industry: A case study on a Danish university"

S1 Overview of AAU data

AAU data show that 109 respondents have switched service providers within the last 36 months. Observing the reasons for churn is found to be that a similar or greater usage package (call hours/GB data) or a similar or cheaper price was the greatest reason for switching providers. Another key factor compelling the respondents to switch was promotional offers included in the package upon switching.

The key factors affecting the respondents' stay are that they are satisfied with their current package, satisfied with the customer service, and/or the provider offered upgrades to their subscription plan. It is found that family and friends have the greatest influence on the switching decision for 36.9% of all respondents, whereas advertisement through social media or other media (TV, radio, internet) accounts for 27% of switched respondents. For instance, five respondents have answered the question, *Which among the following reasons compelled you to take the switching decision?* Their qualitative response was they switched as their parents also switched, and parents are responsible for the subscription. Other factors found were direct advertising through call-centers, a direct offer from a store employee, or the respondent's own price comparison research.

Fig. S1.3 presents the ranking of attributes found relevant for a part of Danish customers when subscribing to a service provider based on respondents' five-point Likert scale answers (1-strongly disagree and 5-strongly disagree).

S1.1 Detailed response of AAU survey for each questions and results

The following figures presents all questions and distribution of answers in percentage.

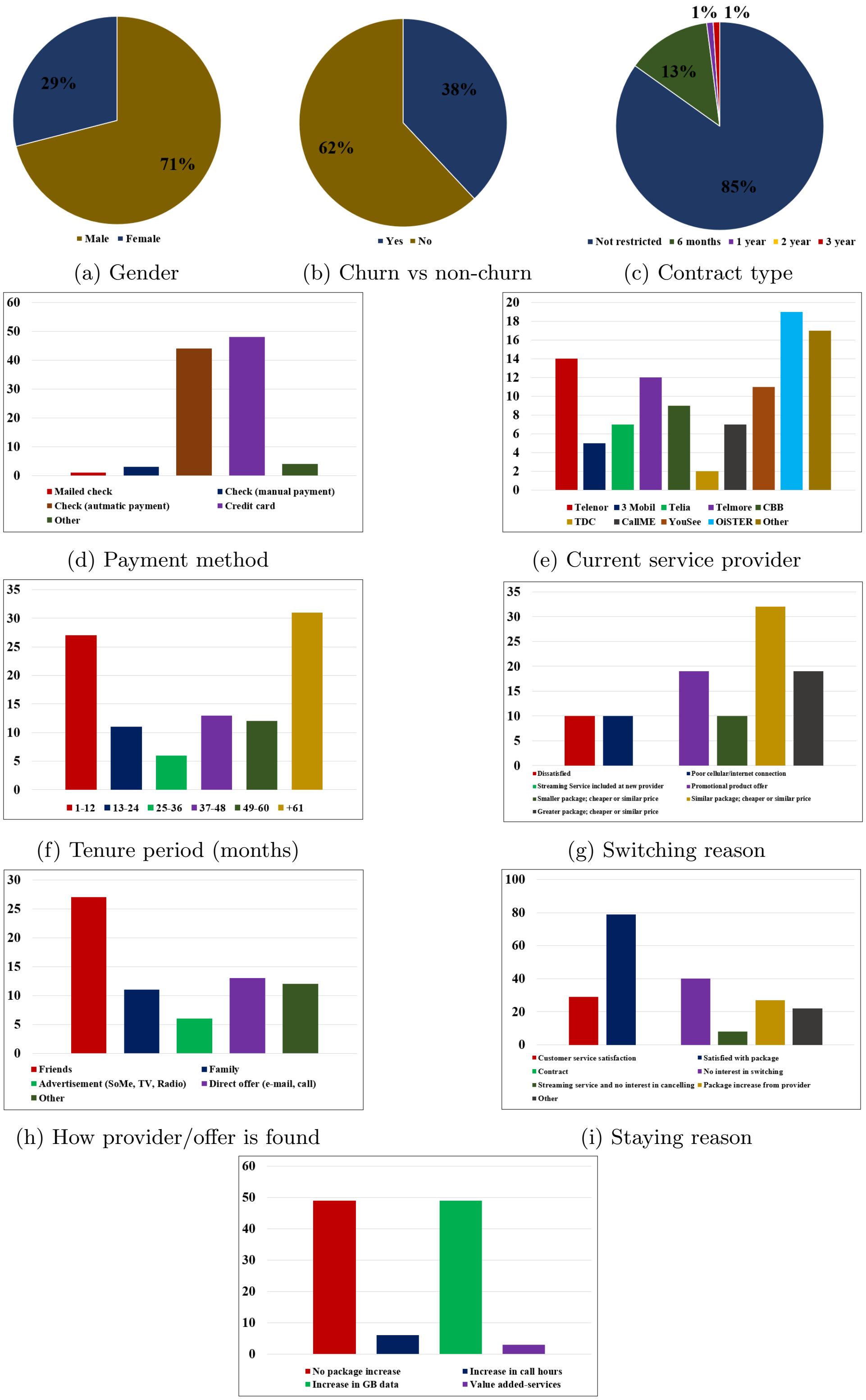

(a) Gender (b) Churn vs non-churn (c) Contract type

(d) Payment method (e) Current service provider

(f) Tenure period (months) (g) Switching reason

(h) How provider/offer is found (i) Staying reason

(j) Subscription plan offers

Fig. S1.1: Overview of responses

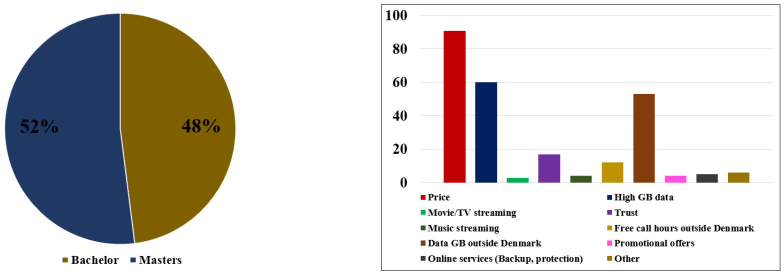

(a) Study (b) Important attributes

Fig. S1.2: Overview of responses

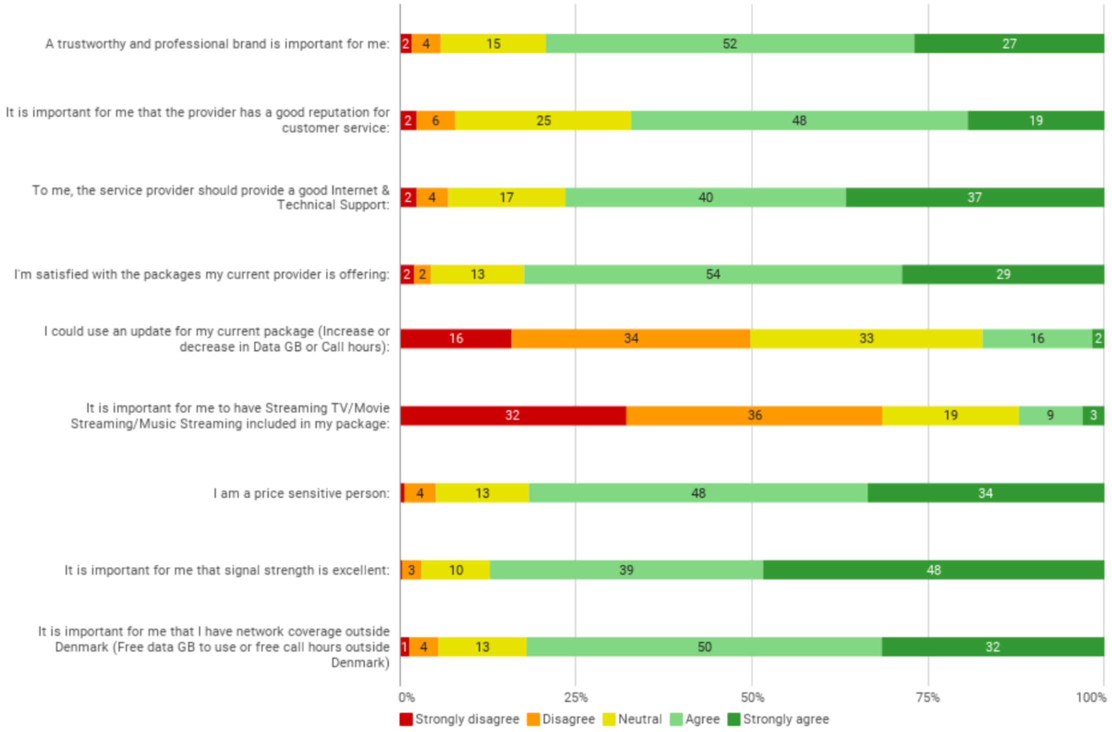

Fig. S1.3: Sensitivity to attributes amongst respondents based on 5-point Likert Scale

S2 Description of features from datasets

Table S2.1: Description of features for IBM telco and Maven telco data [37], [50]

**Feature**	**Dataset**	**Description**
Age	Maven	Age of customer
Gender	Maven & Telco	Gender of the customer
Tenure in Months	Maven & Telco	Total amount of months the customer has been with provider
Offer	Maven	Indicates which specific offer the customer accepted, if any
Phone Service	Maven & Telco	"Yes" or "No" if customer subscribes to phone service
Avg Monthly Long Distance Charges	Maven	The customer's average long distance charges
Multiple Lines	Maven & Telco	"Yes" or "No" if customer is subscribed to multiple lines
Internet Service	Maven & Telco	"Yes" or "No" if customer is subscribed to an internet service
Internet Type	Maven & Telco	Which internet type the customer is connected to
Avg Monthly GB Download	Maven	Customer's average monthly gigabyte(s) download
Online Security	Maven & Telco	"Yes" or "No" if customer is subscribed to additional online security service
Online Backup	Maven & Telco	"Yes" or "No" if customer is subscribed to additional online backup service
Device Protection Plan	Maven & Telco	"Yes" or "No" if customer is subscribed to device protection plan
Premium Tech Support	Maven & Telco	"Yes" or "No" if customer is subscribed to technical support
Streaming TV	Maven & Telco	"Yes" or "No" if customer use internet to stream TV at third party provider (no fees)
Streaming Movies	Maven & Telco	"Yes" or "No" if customer use internet to stream movies at third party provider (no fees)
Streaming Music	Maven & Telco	"Yes" or "No" if customer use internet to stream music at third party provider (no fees)
Unlimited Data	Maven	"Yes" or "No" if customer pay extra fee for unlimited gigabytes for download
Contract	Maven & Telco	Indicates which specific contract the customer have
Paperless Billing	Maven & Telco	"Yes" or "No" if customer choose paperless billing
Payment Method	Maven & Telco	Indicates how the customer pays the bill
Monthly Charge	Maven & Telco	Indicates the customer's total monthly charge for all subscribed services
Total Charges	Maven & Telco	Indicates the customer's total charges (until Q2 2022)
Total Extra Data Charges	Maven	Indicates the customer's total charges for extra data (until Q2 2022)
Total Long Distance Charges	Maven	Indicates the customer's total charges for extra long distance (until Q2 2022)
Total Revenue	Maven	Indicates the customer's total revenue (sum of all charges until Q2 2022)
Customer Status	Maven	"Churned" or "Stayed" indicates the status of customer (Q2 2022)
Churn	Telco	"Yes" or "No" indicates whether customer is churned or not churned

Table S2.2: Description of features after exclusion for Cell2Cell dataset [38]

**Feature**	**Description**
PeakCallsInOut	Number of peak voice calls in and out
ServiceArea	Service area of customer
InboundCalls	Number of inbound voice calls
DroppedBlockedCalls	Number of blocked or dropped calls
ThreewayCalls	Number of three-way calls
UniqueSubs	Number of unique subs
CallWaitingCalls	Number of call waiting calls
HandsetRefurbished	”Yes” or ”No”; if handset is refurbished
CallForwardingCalls	Number of call waiting calls
UnansweredCalls	Number of unanswered voice calls
OverageMinutes	Overage minutes of use
RetentionOffersAccepted	Number of previous retention offers accepted
ReferralsMadeBySubscriber	Number of referrals made by subscriber
NonUSTravel	”Yes” or ”No”; has traveled to a non-US country
NewCellphoneUser	”Yes” or ”No”; known to be a new cell phone user
CustomerCareCalls	Number of customer care calls
PercChangeRevenues	Percentage change in revenue
HandsetWebCapable	”Yes” or ”No”; handset is web capable
IncomeGroup	Which income group the customer belongs to
NotNewCellphoneUser	”Yes” or ”No”; known to not be a new cell phone user
DirectorAssistedCalls	Number of director assisted calls
RetentionCalls	Number of previously calls a customer has made to retention team
MonthsInService	Number of months in service (tenure)
MadeCallToRetentionTeam	”Yes” or ”No” a customer has made call to retention team
ActiveSubs	Number of active subs
OptOutMailings	”Yes” or ”No” whether customer request mailings
RespondsToMailOffers	”Yes” or ”No” if customer responds to mail offers
RoamingCalls	Number of roaming voice calls
Handsets	Number of handsets issued
OffPeakCallsInOut	Number of in and out-off peak voice calls
TotalRecurringCharge	Total recurring charge
OutboundCalls	Number of outbound voice calls
DroppedCalls	Number of dropped voice calls
CurrentEquipmentDays	Number of days of the current equipment
ReceivedCalls	Minutes of use for received voice calls
MonthlyRevenue	Average monthly revenue
PercChangeMinutes	Percentage change in minutes used
MonthlyMinutes	Average monthly minutes of use
Churn	”Yes” or ”No” indicates whether customer is churned or not churned

S3 MLAs performance results & parameter settings

Table S3.1: Performance analysis of classification algorithms

Method	Accuracy	F1-Score	Precision	AUC	NPV
Telco					
LR	**0.796**	**0.578**	0.650	**0.709**	**0.836**
XGBC	0.774	0.539	0.595	0.685	0.825
ADA	0.789	0.568	0.630	0.703	0.834
DT	0.707	0.474	0.456	0.639	0.809
RF	0.793	0.534	**0.671**	0.682	0.819
Maven					
LR	0.774	0.664	0.705	0.741	0.806
XGBC	**0.831**	**0.751**	0.791	**0.805**	**0.850**
ADA	0.818	0.737	0.759	0.796	0.848
DT	0.791	0.701	0.712	0.768	0.832
RF	0.817	0.713	**0.805**	0.777	0.821
Cell2Cell					
LR	0.709	0.008	0.257	0.500	0.711
XGBC	**0.717**	0.272	0.527	**0.558**	**0.738**
ADA	0.714	0.158	0.526	0.529	0.724
DT	0.617	**0.343**	0.340	0.537	0.733
RF	**0.717**	0.150	**0.567**	0.530	0.724
AAU					
LR	0.644	0.311	0.389	0.538	0.710
XGBC	0.908	0.846	**0.880**	0.882	0.919
ADA	0.920	0.873	0.857	0.911	0.949
DT	0.874	0.792	0.808	0.847	0.902
RF	**0.931**	**0.893**	0.862	**0.930**	**0.966**

The results in Table S3.1 reflect that a single algorithm cannot ensure higher performance measure with respect to all datasets. For example XGBC perform good in Maven dataset, but not in Telco dataset. Therefore, if we rely on XGBC only, we might miss some of the key features that are identified by other best-performing algorithms.

Table S3.2: Parameter settings used for machine learning algorithms

Algorithm	Reference	Parameter set
Random forest	[34]	bootstrap = *true*, ccp_alpha = 0.0, criterion = *gini*, max_depth = none, max_features = *auto*, min_samples_split = 2 max_leaf_nodes = 30, n_estimators = 200-500, oob_score = *true*
AdaBoost	[34]	algorithm = *SAMME.R*, base_estimator = *none* learning_rate = 1.0, n_estimators = 50-200, random_state = *none*
Logistic regression	[34]	fit_intercept = *true*, intercept_scaling = 1.0, l1_ratio = *none*, max_iter = 100, penalty = *l2*, solver = *lbfgs*, tolerance = 0.0001, verbose = 0
XGBC	[72]	objective = *binary*, base_score = 0.5, booster = *gbtree*, grow˙policy = *depthwise*, learning_rate = 0.300000012, max_bins = 256, max_depth = 6-12, n_estimators = 100-200, predictor = *auto*, sampling_method = *uniform*, tree_method = *exact*
Decision tree	[34]	criterion = *gini*, max_depth = 10-*none*, min_samples_split = 2 min_samples_leaf = 1, splitter = *best*, max_features = *none*

S3.1 Feature importance for five MLAs

Table S3.3: Permutation importance of top five features affecting customer churn

Rank	LR	XGBC	ADA	DT	RF
Telco					
1	Tenure	Tenure	Tenure	Contract	Tenure
2	MonthlyCharges	Contract	MonthlyCharges	MonthylCharges	Contract
3	Contract	MonthlyCharges	Contract	Tenure	InternetService
4	PhoneService	OnlineSecurity	OnlineSecurity	MultipleLines	MonthlyCharges
5	InternetService	InternetServices	TechSupport	OnlineSecurity	TechSupport
Maven					
1	Contract	Contract	Tenure in Months	Tenure in Months	Contract
2	Total Charges	Tenure in Months	Contract	Contract	Monthly Charge
3	Monthly Charge	Age	Monthly Charge	Monthly Charge	Age
4	Avg. Monthly GB	Monthly Charge	Online Security	Total Charges	Premium Tech Support
5	Avg Mon. Long Distance Charges	Online Security	Avg. Monthly GB	Total Long Distance Charges	Multiple Lines
Cell2Cell					
1	PercChangeRevenues	MonthlyMinutes	MonthlyMinutes	MonthlyRevenue	PercChangeMinutes
2	PercChangeMinutes	PercChangeMinutes	PercChangeMinutes	CurrentEquipmentDays	MonthlyMinutes
3	MonthlyMinutes	MonthlyRevenue	CurrentEquipmentDays	PercChangeMinutes	MonthsInService
4	OffPeakCallsInOut	MonthsInService	MonthsInService	TotalRecurringCharge	PeakCallsInOut
5	OverageMinutes	CurrentEquipmentDays	RetentionCalls	MonthlyMinutes	CurrentEquipmentDays
AAU					
1	TechSupport	PackageIncrease	PackageIncrease	PackageIncrease	PackageIncrease
2	PackageSatisfaction	TechSupport	Streaming-TV-Movie-Music	ImportantCriteria	Contract
3	Brand	Price	PaymentMethod	Streaming-TV-Movie-Music	Price
4	Gender	CustomerService	CustomerService	PaymentMethod	TechSupport
5	Streaming-TV-Movie-Music	PackageSatisfaction	NetworkCoverage	NetworkCoverage	NetworkCoverage

Table S3.4: Feature importance results for AAU Survey

Feature	XGBC	ADA	DT	RF	LR
Brand	-0.0069	0.0000	-0.0092	0.0000	0.0092
Contract	0.0023	0.0000	0.0000	0.0253	-0.0023
CurrentPackageUpdate	0.0000	0.0000	0.0000	0.0046	-0.0092
CustomerService	0.0230	0.0069	-0.0115	0.0000	0.0000
Gender	-0.0046	0.0000	0.0000	-0.0023	0.0046
NetworkCoverage	0.0046	0.0046	0.0023	0.0092	-0.0207
PackageIncrease	0.3333	0.3609	0.2621	0.3172	-0.0161
PackageSatisfaction	0.0092	0.0023	0.0000	0.0092	0.0092
PaymentMethod	-0.0023	0.0092	0.0046	0.0000	0.0000
Price	0.0230	-0.0023	-0.0046	0.0161	-0.0138
Signal	-0.0046	0.0000	-0.0023	0.0023	-0.0115
StreamingTV˙Music˙Movie	0.0023	0.0207	0.0069	0.0069	0.0023
Study	0.0046	0.0000	0.0000	-0.0046	-0.0023
TechSupport	0.0253	-0.0023	0.0009	0.0115	0.0184
Tenure	-0.0023	0.0000	0.0000	0.0000	-0.0092

Table S3.5: Feature importance results for IBM Telco dataset

Feature	XGBC	ADA	DT	RF	LR
Contract	0.0242	0.0233	0.0281	0.0277	0.0186
Dependents	-0.0006	0.0000	0.0006	0.0010	-0.0004
DeviceProtection	-0.0003	-0.0009	0.0009	-0.0017	0.0001
Gender	-0.0012	0.0000	-0.0067	-0.0005	0.0009
InternetService	0.0079	0.0000	0.0024	0.0094	0.0075
MonthlyCharges	0.0094	0.0336	0.0101	0.0087	0.0522
MultipleLines	0.0016	-0.0007	0.0040	0.0027	0.0036
OnlineBackup	0.0010	0.0026	0.0016	0.0034	0.0010
OnlineSecurity	0.0084	0.0074	0.0035	0.0064	0.0019
PaperlessBilling	0.0039	0.0002	-0.0073	0.0044	0.0027
PaymentMethod	0.0012	-0.0004	-0.0055	0.0049	0.0009
PhoneService	0.0000	0.0001	0.0006	-0.0002	0.0134
SeniorCitizen	0.0052	0.0009	0.0004	0.0011	-0.0015
StreamingMovies	-0.0003	0.0000	0.0024	-0.0005	0.0002
StreamingTV	0.0028	0.0000	-0.0001	-0.0001	-0.0012
TechSupport	0.0078	0.0034	-0.0014	0.0067	0.0038
Tenure	0.0451	0.0469	0.0090	0.0387	0.0523
TotalCharges	-0.0050	-0.0020	-0.0054	0.0016	0.0028

Table S3.6: Feature importance results for Maven Telco dataset

Feature	XGBC	ADA	DT	RF	LR
Age	0.0278	0.0033	0.0139	0.0072	0.0016
Avg Monthly GB Download	0.0001	0.0046	-0.0019	-0.0007	0.0098
Avg Monthly Long Distance Charges	0.0020	0.0001	-0.0055	-0.0006	0.0083
Contract	0.0854	0.0458	0.0724	0.0417	0.0514
Device Protection PLan	0.0023	0.0000	-0.0019	-0.0001	0.0029
Gender	0.0028	0.0000	0.0003	-0.0001	-0.0001
Internet Service	0.0000	0.0000	0.0000	0.0000	0.0000
Internet Type	0.0030	0.0010	0.0041	0.0008	-0.0033
Monthly Charge	0.0185	0.0242	0.0368	0.0109	0.0132
Multiple Lines	0.0028	0.0000	0.0022	0.0017	0.0035
Offer	0.0014	0.0013	0.0020	-0.0009	0.0004
Online Backup	-0.0014	-0.0022	0.0004	-0.0001	0.0014
Online Security	0.0049	0.0054	-0.0007	-0.0006	0.0020
Paperless Billing	0.0013	0.0016	0.0051	-0.0003	0.0061
Payment Method	0.0037	0.0028	0.0003	0.0010	0.0041
Phone Service	0.0000	0.0000	0.0000	0.0000	0.0000
Premium Tech Support	-0.0004	0.0020	0.0007	0.0019	0.0048
Streaming Movies	-0.0003	0.0000	-0.0030	-0.0020	0.0006
Streaming Music	0.0019	0.0000	-0.0019	-0.0007	0.0007
Streaming TV	0.0012	0.0000	0.0007	-0.0012	-0.0003
Tenure in Months	0.0310	0.0717	0.0914	-0.0025	0.0042
Total Charges	0.0020	-0.0035	0.0274	-0.0033	0.0348
Total Extra Data Charges	0.0001	0.0000	0.0000	0.0001	0.0007
Total Long Distance Charges	0.0035	-0.0039	0.0168	-0.0087	0.0020
Total Revenue	-0.0006	0.0004	0.0068	-0.0051	0.0001
Unlimited Data	-0.0009	0.0000	-0.0003	-0.0003	-0.0003

Table S3.7: Feature importance results for Cell2Cell dataset

Feature	XGBC	ADA	DT	RF	LR
ActiveSubs	0.0010	0.0000	-0.0003	-0.0003	-0.0001
RoamingCalls	0.0008	0.0000	0.0048	0.0004	0.0004
CallForwardingCalls	0.0001	0.0000	-0.0001	0.0000	0.0000
CallWaitingCalls	0.0016	0.0000	0.0032	0.0017	0.0000
CurrentEquipmentDays	0.0114	0.0041	0.0237	0.0026	0.0001
CustomerCareCalls	0.0030	0.0005	0.0026	0.0013	0.0004
DirectorAssistedCalls	0.0010	0.0002	0.0022	0.0002	0.0000
DroppedBlockedCalls	0.0028	0.0000	0.0138	0.0009	0.0000
DroppedCalls	0.0030	-0.0005	0.0097	0.0005	0.0002
HandsetRefurbished	0.0000	-0.0001	-0.0006	-0.0005	0.0000
Handsets	0.0008	0.0000	0.0013	-0.0003	0.0002
HandsetWebCapable	0.0008	0.0005	0.0006	0.0007	0.0000
InboundCalls	0.0040	0.0005	0.0064	0.0010	0.0002
IncomeGroup	-0.0004	-0.0013	-0.0010	0.0005	0.0000
MadeCallToRetentionTeam	0.0000	0.0000	0.0004	0.0003	0.0000
MonthlyMinutes	0.0435	0.0093	0.0220	0.0080	0.0010
MonthlyRevenue	0.0159	-0.0012	0.0255	0.0008	-0.0003
MonthsInService	0.0126	0.0030	0.0219	0.0034	0.0000
NewCellphoneUser	0.0000	0.0000	-0.0001	-0.0005	0.0000
NonUSTravel	0.0001	0.0000	-0.0004	0.0002	0.0000
NotNewCellphoneUser	0.0003	0.0000	0.0009	0.0002	0.0000
OffPeakCallsInOut	0.0075	0.0000	0.0138	0.0020	0.0008
OptOutMailings	0.0001	0.0000	-0.0004	-0.0001	0.0000
OutboundCalls	0.0025	0.0000	0.0093	0.0010	0.0001
OverageMinutes	0.0025	-0.0009	0.0079	0.0025	0.0006
PeakCallsInOut	0.0068	0.0000	0.0096	0.0031	0.0006
PercChangeMinutes	0.0331	0.0047	0.0233	0.0125	0.0020
PercChangeRevenues	0.0051	0.0015	0.0131	0.0002	0.0018
ReceivedCalls	0.0054	0.0004	0.0139	0.0010	-0.0004
ReferralsMadeBySubscriber	-0.0003	0.0000	0.0001	-0.0003	0.0000
RespondsToMailOffers	0.0012	-0.0005	0.0003	0.0011	0.0000
RetentionCalls	0.0010	0.0019	0.0006	0.0001	0.0000
RetentionOffersAccepted	-0.0001	0.0000	0.0000	-0.0002	0.0000
ServiceArea	0.0021	0.0000	0.0029	0.0006	-0.0004
ThreewayCalls	0.0005	0.0000	0.0009	0.0005	0.0001
TotalRecurringCharge	0.0100	0.0003	0.0223	0.0004	-0.0006
UnansweredCalls	0.0027	0.0000	0.0032	0.0004	0.0000
UniqueSubs	0.0040	0.0015	0.0007	0.0015	0.0000

S4 Top-decile lift results

Table S4.1: Lift result for AAU data

Decile	No. of cases	No. of responses	Cumulative responses	% of events	Gain	Lift
Random forest						
1	9	5	5	18.52	18.52	1.85
2	9	3	8	11.11	29.63	1.48
3	8	2	10	7.41	37.04	1.23
4	9	2	12	7.41	44.45	1.11
5	8	0	12	0	44.45	0.89
6	9	0	12	0	44.45	0.74
7	9	4	16	14.81	59.26	0.85
8	8	4	20	14.81	74.07	0.93
9	7	3	23	11.11	85.18	0.95
10	11	4	27	14.81	99.99	1
XGBClassifier						
1	9	5	5	18.52	18.52	1.85
2	9	3	8	11.11	29.63	1.48
3	8	2	10	7.41	37.04	1.23
4	9	2	12	7.41	44.45	1.11
5	8	0	12	0	44.45	0.89
6	9	0	12	0	44.45	0.74
7	9	4	16	14.81	59.26	0.85
8	8	4	20	14.81	74.07	0.93
9	9	3	23	11.11	85.18	0.95
10	9	4	27	14.81	99.99	1

Table S4.2: Lift result for IBM telco data

Decile	No. of cases	No. of responses	Cumulative responses	% of events	Gain	Lift
Random forest						
1	212	63	63	11.13	11.13	1.11
2	211	57	120	10.07	21.2	1.06
3	211	59	179	10.42	31.62	1.05
4	211	60	239	10.6	42.22	1.06
5	211	57	296	10.07	52.29	1.05
6	212	52	348	9.19	61.48	1.02
7	211	55	403	9.72	71.2	1.02
8	211	55	458	9.72	80.92	1.01
9	211	61	519	10.78	91.7	1.02
10	212	47	566	8.3	100	1
Logistic regression						
1	212	63	63	11.13	11.13	1.11
2	211	57	120	10.07	21.2	1.06
3	211	59	179	10.42	31.62	1.05
4	211	60	239	10.6	42.22	1.06
5	211	57	296	10.07	52.29	1.05
6	212	52	348	9.19	61.48	1.02
7	211	55	403	9.72	71.2	1.02
8	211	55	458	9.72	80.92	1.01
9	211	61	519	10.78	91.7	1.02
10	212	47	566	8.3	100	1

Table S4.3: Lift result for Maven telco data

XGBClassifier						
Decile	No. of cases	No. of responses	Cumulative responses	% of events	Gain	Lift
1	138	55	55	11.2	11.2	1.12
2	138	48	103	9.78	20.98	1.05
3	138	56	159	11.41	32.39	1.08
4	138	56	215	11.41	43.8	1.1
5	138	44	259	8.96	52.76	1.06
6	138	56	315	11.41	64.17	1.07
7	138	40	355	8.15	72.32	1.03
8	138	42	397	8.55	80.87	1.01
9	138	47	444	9.57	90.44	1
10	139	47	491	9.57	100.01	1
Random forest						
1	138	55	55	11.2	11.2	1.12
2	134	48	103	9.78	20.98	1.05
3	136	54	157	11	31.98	1.07
4	133	54	211	11	42.98	1.07
5	134	44	255	8.96	51.94	1.04
6	142	58	313	11.81	63.75	1.06
7	142	40	353	8.15	71.9	1.03
8	115	34	387	6.92	78.82	0.99
9	148	48	435	9.78	88.6	0.98
10	159	56	491	11.41	100.01	1

Table S4.4: Lift result for Cell2Cell data

XGBClassifier						
Decile	No. of cases	No. of responses	Cumulative responses	% of events	Gain	Lift
1	1493	447	447	10.29	10.29	1.03
2	1492	398	845	9.16	19.45	0.97
3	1493	418	1263	9.62	29.07	0.97
4	1492	404	1667	9.3	38.37	0.96
5	1493	444	2111	10.22	48.59	0.97
6	1492	485	2596	11.17	59.76	1
7	1493	402	2998	9.26	69.02	0.99
8	1492	453	3451	10.43	79.45	0.99
9	1493	451	3902	10.38	89.83	1
10	1493	441	4343	10.15	99.98	1
Random forest						
1	1342	396	396	9.12	9.12	0.91
2	1312	360	756	8.29	17.41	0.87
3	1501	415	1171	9.56	26.97	0.9
4	1769	481	1652	11.08	38.05	0.95
5	1469	439	2091	10.11	48.16	0.96
6	1466	482	2573	11.1	59.26	0.99
7	1352	364	2937	8.38	67.64	0.97
8	1606	472	3409	10.87	78.51	0.98
9	1462	455	3864	10.48	88.99	0.99
10	1647	479	4343	11.03	100.02	1
Decision tree						
1	1417	422	422	9.72	9.72	0.97
2	1557	418	840	9.62	19.34	0.97
3	1448	405	1245	9.33	28.67	0.96
4	1146	316	1561	7.28	35.95	0.9
5	1581	460	2021	10.59	46.54	0.93
6	1782	567	2588	13.06	59.6	0.99
7	1202	331	2919	7.62	67.22	0.96
8	1799	530	3449	12.2	79.42	0.99
9	1045	319	3768	7.35	86.77	0.96
10	1949	575	4343	13.24	100.01	1

S5 Balanced AAU dataset: Performance and feature importance results

Table S5.1: Performance analysis of classification algorithms: Balanced AAU dataset by using SMOTE

AAU data					
Method	Accuracy	F1-Score	Precision	AUC	NPV
LR	0.607	0.607	0.600	0.607	0.577
XGBC	0.953	0.954	0.960	0.954	0.941
ADA	0.879	0.875	0.840	0.876	0.894
DT	0.925	0.923	0.900	0.924	0.938
RF	0.953	0.952	0.940	0.952	0.959

Table S5.2: Permutation importance of top five features affecting churn on AAU dataset: Best-performing algorithm based on balanced dataset by using SMOTE

AAU data				
Rank	RF		XGBC	
1	PackageIncrease	0.3850	PackageIncrease	0.4019
2	Price	0.0280	Price	0.0224
3	Contract	0.0206	NetworkCoverage	0.0187
4	NetworkCoverage	0.0112	PaymentMethod	0.0187
5	PaymentMethod	0.0112	TechSupport	0.0187
